# Harnessing Mesenchymal Stromal Cells for Advanced Wound Healing: A Comprehensive Review of Mechanisms and Applications

**DOI:** 10.3390/ijms26010199

**Published:** 2024-12-29

**Authors:** Khrystyna Nasadiuk, Tomasz Kolanowski, Cezary Kowalewski, Katarzyna Wozniak, Tomasz Oldak, Natalia Rozwadowska

**Affiliations:** 1 Research and Development Department, Polski Bank Komórek Macierzystych S.A. (FamiCord Group), 00-867 Warsaw, Poland; khrystyna.nasadiuk@pbkm.pl (K.N.); tomasz.kolanowski@igcz.poznan.pl (T.K.); 2 Institute of Human Genetics, Polish Academy of Sciences, 60-479 Poznan, Poland; 3 Department of Dermatology, National Medical Institute of the Ministry of the Interior and Administration, 02-507 Warsaw, Poland; czarekkowalewski@gmail.com (C.K.); kwozniak@wum.edu.pl (K.W.)

**Keywords:** mesenchymal stromal cells (MSCs), chronic wounds, burns, diabetic wounds, non-union bone fractures, epidermolysis bullosa

## Abstract

Chronic wounds and injuries remain a substantial healthcare challenge, with significant burdens on patient quality of life and healthcare resources. Mesenchymal stromal cells (MSCs) present an innovative approach to enhance tissue repair and regeneration in the context of wound healing. The intrinsic presence of MSCs in skin tissue, combined with their roles in wound repair, ease of isolation, broad secretory profile, and low immunogenicity, makes them especially promising for treating chronic wounds. This review explores the current landscape of MSC application, focusing on preclinical and clinical data across chronic wounds, diabetic ulcers, burns, non-union bone fractures, lower extremity venous ulcers, pressure ulcers, and genetic skin conditions like epidermolysis bullosa. Special emphasis is given to the mechanisms through which MSCs exert their regenerative effects, underscoring their potential in advancing wound healing therapies and supporting the broader field of regenerative medicine.

## 1. Introduction

Wounds and injuries are probably the first and one of the most prevalent diseases that humanity has encountered since ancient times, and despite the unique capacity of human tissues, regeneration remains an enormous challenge for modern medicine. Chronic wounds occur when there is a failure of injured skin to proceed through an orderly and timely process to produce anatomical and functional integrity and persist as a significant healthcare problem, particularly due to an increasing number of patients and the lack of efficient treatments [1,2,3,4,5,6].

It is estimated that about 500,000 patients in Poland and 4.6 mln in USA are suffering from chronic wounds that affect about 1.5% of the population in industrially developed countries, and about 3% of people are older than 60 years of age; moreover, 50% of those wounds do not respond to current treatments [7,8].

Tissue repair and regeneration after damage are not completely understood, and current therapies to support this process are limited. Thus, the treatment of chronic wounds is a significant concern for global healthcare, and, given the rise in diabetic and aging populations, this medico-economic disease burden will continue to rise [3,9,10]. For instance, in the United States, the treatment of chronic wounds costs more than $25 billion annually [1].

Cell therapy offers an alternative approach for enhancing tissue repair and regeneration following injury, thus ATMP therapies (advanced therapy medicinal product) are emerging as a powerful technique to improve skin wound healing [2,3,5,9]. The presence of mesenchymal stromal cells in skin tissues, coupled with their integral roles in normal wound healing along with ease of isolation and secretory potential, implies that MSCs may be beneficial for chronic wound healing [1,9]. With an increasing understanding of the repair mechanisms, MSCs-based therapy has progressed from animal studies to clinical trials [1,11]. As of 20 September 2024, the National Library of Medicine database included four recruiting clinical trials applying MSCs for the treatment of wounds of different origin, the repair of different musculoskeletal injuries, and one for epidermolysis bullosa patients as well as twenty-eight completed clinical trials, with five of them on epidermolysis bullosa (Table 1 and Table 2).

MSCs are a heterogeneous group of self-renewing multipotent cells arising from a variety of human tissues (bone marrow, umbilical cord and umbilical cord blood, placenta, fat, peripheral circulation, skin, synovium, cartilage, periosteum, vessel walls, muscle, tendon, and dental tissues) [1,3,11,12,13,14,15,16]. In addition to their potential for differentiation into various lineages, including osteogenic, chondrogenic, and adipogenic origin, MSCs can regulate immune and inflammatory processes through paracrine signaling [3,11,12,15,16].

In this review, we summarize the up-to-date current application of mesenchymal stromal cells in the treatment of wounds, diabetic foot ulcers, burns, epidermolysis bullosa, and non-union fractures.

## 2. Mechanisms of Therapeutic Effect of MSCs in Wound Healing

**Figure 1:**
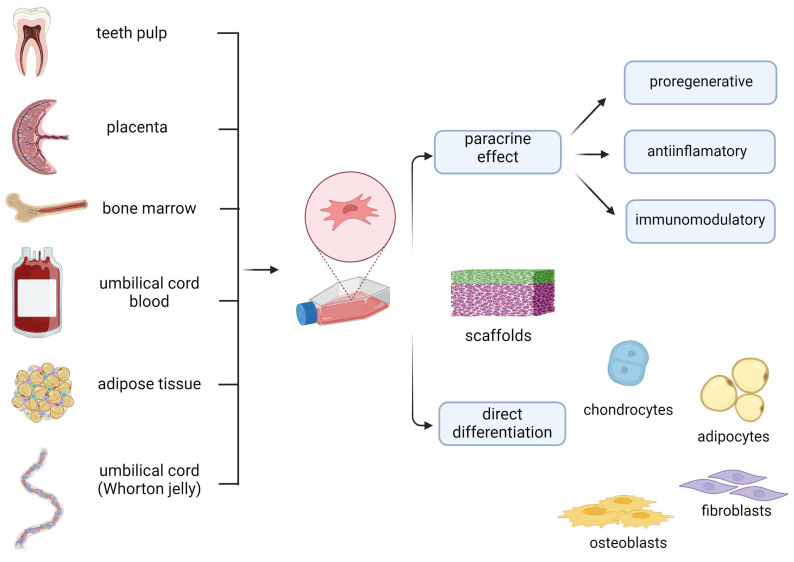
The mechanism of therapeutic effect of MSCs in wound healing.

**Figure 2:**
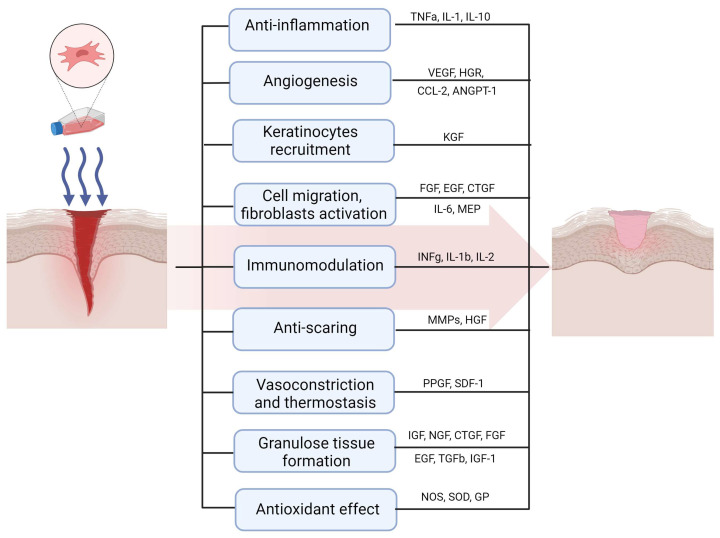
Paracrine effects of MSCs in wound healing.

The normal wound healing process occurs in a regular cellular consequence in response to injury and involves four phases: (1) homeostasis/coagulation, (2) inflammatory cell recruitment, (3) proliferative phase, and (4) maturation phase [3,6,10,11,15,17,18,19,20]. Chronic wounds are those that fail to progress through the normal stages of healing, resulting in a tissue injury that is not repaired within the typical period, and wound healing is often prevented by high levels of inflammatory factors [1,8]. MSCs that are normal constituents of skin homeostasis and physiological processes of wound healing [9] were shown to enhance regeneration processes through the following mechanisms (Figure 1 and Figure 2):

- Secretion of paracrine factors (promoting epithelialization, angiogenesis, granulation tissue formation, and the activation of fibroblasts) [5,9,10,11,16,18,20];
- Immunomodulatory and anti-inflammatory [1,5,9,10];
- Skin cells recruitment [1,5,21];
- Antimicrobial effect (enhancing the ability of immune cells to kill bacteria and to secrete antimicrobial peptides, such as LL-37 [1], as well as possible direct bactericidal activity shown in vitro [22].

The evidence shows that MSCs can potentially affect and improve every stage of wound healing [9,17,23]. In the inflammatory phase, MSCs produce biologically active substances, affecting the activity of immune cells and myofibroblasts, which lead to the decrease in TNFα, the increase in IFNγ secretion, and enhance wound contraction. MSCs could trigger the release of anti-inflammatory IL4. During the proliferative phase, the application of MSC was shown to enhance the survival and migration of fibroblasts, resulting in increased deposition of extracellular matrix. In the maturation phase, paracrine factors released by MSCs exert anti-fibrotic effects [17]. Skin regeneration following direct MSCs implementation into a wound was reported to be characterized by a reduction in scar formation [22]. MSCs were also shown to promote a regenerative, rather than fibrotic, wound healing microenvironment [9].

MSCs have been found to differentiate into the cells of damaged tissues in many animal models, such as diabetic skin ulcers, and osteoarthritis; furthermore, they can migrate into distant injured tissues to facilitate tissue repair [1]. MSCs regulate immune response and inflammation and possess powerful tissue-protective and reparative mechanisms through the secretion of numerous paracrine factors (e.g., growth factors and cytokines) to improve the survival, proliferation, migration, and differentiation of cells involved in wound re-epithelialization [1,8,24,25,26]. The limited engraftment of MSCs, along with their reduced persistence and survival in the microenvironment of chronic wounds, supports the theory that MSCs exert their beneficial effects on skin wound healing primarily through the secretion of trophic factors in a paracrine or autocrine manner [1,18].

All types of cutaneous wound healing require de novo angiogenesis for the transportation of systematic oxygen, nutrients, and other necessities into proximal wounded sites during the entire tissue repairing process [21,27]. The angiogenic conditions in physiological and impaired wound healing are different. Normal wound healing is characterized by the increased secretion of angiogenic factors from the moment of injury with the highest level before maximum capillary formation, whereas in chronic wounds, the effect of anti-angiogenic molecules (e.g., increased levels of matrix metalloproteinases) is dominating [1]. MSCs have been shown to facilitate wound healing by increasing angiogenesis, inhibiting inflammation, and promoting the migration of fibroblasts and collagen production through paracrine mechanisms [9,18,28]. Soluble factors secreted by MSCs reinforce endogenous mechanisms for tissue repair and regeneration by recruiting and improving native cell activity in the target tissue [11,18]. MSCs enhance angiogenesis in chronic wounds, mainly the secretion of various bioactive factors, including exosomes, growth factors, and chemokines, which facilitates new blood vessel formation by stimulating the recruitment and proliferation of host endothelial cells [1]. MSCs may contribute to neovascularization in adults via the release of proangiogenic factors such as HIF-1, VEGF, EGF, and CXCL12 [17]. The modulation of angiogenesis to improve wound healing has been explored in numerous studies. VEGF, the principal angiogenic factor, is the most potent mitogen and is found in the endothelial cells of arterial, venous, and lymphatic vessels [21]. Recent studies have demonstrated that, in addition to endothelial cells, multiple cell types involved in the wound healing process express VEGF, and their ligand–receptor interactions play a critical role in initiating angiogenesis. The concept of a VEGF-driven response in keratinocytes offers a novel perspective for advancing wound healing therapies [4]. Shabbir et al. reported that MSC exosomes also appeared to induce changes via the activation of growth factor signaling cascades in target cells, including AKT, STAT3, and ERK. By stimulating these pathways, target cells would increase their expression of a number of growth factors, namely HGF, IL-6, IGF1, NGF, and SDF1 [16].

Due to their immunomodulation activities, MSCs help overcome the hyperinflammation of chronic wounds, which promotes the transition of wound healing from the inflammatory phase to the proliferative phase [1]. When activated by inflammatory factors, MSCs were shown to suppress T cell proliferation, B lymphocyte differentiation, and NK cell functions; also, they modulate macrophage phenotypes, disturb dendritic cell functions, and decrease the secretion of pro-inflammatory cytokines [1]. On a rat model of cutaneous wound transplantations with human umbilical cord, MSCs significantly decreased the number of inflammatory cells and pro-inflammatory molecules (IL-1 and TNF-α) [15].

Soluble factors secreted by MSCs were shown to inhibit Staphylococcus aureus biofilm formation in vitro and disrupt the growth of established biofilms along with synergistic killing of drug-resistant bacteria when combined with several major classes of antibiotics. The antimicrobial activity of MSCs was confirmed also in vivo; systemic administration of MSCs to mice with established S. aureus biofilm infections significantly reduced bacterial numbers at the wound site and improved wound healing when combined with antibiotic therapy [22]. MSCs have also been shown to have significant antioxidative properties, protecting against ischemia–reperfusion-induced vascular damage, hypoxia, oxidative DNA damage, and apoptosis in vivo [20].

For experimental and clinical research, MSCs are most commonly isolated from the bone marrow, adipose tissue, Wharton jelly, placenta, and umbilical cord blood [5,19,21,28,29]. In experimental studies, MSCs from different sources were shown to improve wound closure [19]. Among other properties, Wharton jelly MSCs have demonstrated higher proliferative capacity with no signs of senescence over serial passages compared to bone marrow MSCs, with lower immunogenicity than their adult tissue counterparts, and they possess potent immunomodulatory properties [5]. Among the various sources for obtaining MSCs, extraembryonic tissues like umbilical cord and Wharton jelly are considered advantageous due to their feasibility, cost-effectiveness, non-invasive collection process, and lack of ethical concerns [5,21]. The fact that the regenerative abilities of MSCs can be altered with age also supports the advantages of perinatal tissues as MSC sources [27].

Traditionally, most studies have used the technique of injecting MSCs intradermally into or around the wound area [15], but based on the inherent ability of MSCs to migrate into injured skin, systemic cell delivery methods have also been widely employed [1]. However, due to the low engraftment of injected MSCs at the wounds site, both in local and systemic administration, different MSCs delivery vehicles and other strategies to enhance cells engraftment are being developed [1,15]. The latter include namely the application of cell-seeded matrixes, scaffolds, fibrin spray matrix, fibrin mesh scaffold, and the simultaneous administration of growth factors [1,15,30].

## 3. Chronic Wounds

Chronic wounds are defined as wounds that do not heal within three months, usually accompanied by persistent inflammatory condition and neutrophils infiltration [1,2,3,4,5]. Most chronic wounds can be categorized into arterial, venous or diabetic ulcers, and pressure sores [1]. Since diabetic wounds have more complex pathogenesis with contributions of hyperglycemia, impaired vascularization, and neuropathy, we reviewed them in a separate section.

The physiological process of cutaneous wound healing involves complex molecular and cellular mechanisms, including cellular proliferation, migration, angiogenesis, the development of extracellular matrix, and tissue remodeling [16,18]. Impairment in one or several of these processes may cause wound chronicity [18]. A highly evolved fibroproliferative response to injury that quickly restores the skin barrier, thereby reducing the risk of infection and further injury, is also essential for orderly cutaneous wound repair [3]. Chronic non-healing wounds are characterized by the decreased production of growth factors and chemokines, reduced angiogenesis, decreased proliferation, the reduced migration of fibroblasts, and high levels of inflammatory factors [1,15,16].

MSCs represent a source of autologous cell-based therapy for chronic wounds due to their multi-regenerative potential [27]. Low immunogenicity arising from the lack of the major histocompatibility complex class II or costimulatory molecules (CD80, CD40, and CD86) also makes MSCs an attractive candidate for allogeneic therapy [1,11,20]. As mentioned above, the main beneficial effects of MSCs in wound healing are regenerative immunomodulatory, anti-inflammatory, and antimicrobial. The enhancement of wound healing by MSCs was observed in a variety of animal models and in reported clinical cases [8]. Factors secreted by MSCs have been shown to enhance chronic wound healing in vitro and in vivo [31]. Besides VEGF, Malhotra et al. distinguished the following growth factors that modulate re-epithelization, angiogenesis, hemotaxis, tissue granulation, and wound closure, namely, angiopoietin, connective tissue growth factors, epidermal growth factor, fibroblast growth factors, insulin-like growth factors, keratinocyte growth factor, nerve growth factor, platelet-derived growth factor, transforming growth factor, and hepatocyte growth factor [32]. Human MSCs culture media were shown to contain IL-5, IL-6, IL-8, IL-9, IP-10, MCP-1, FGF-2, and VEGF [31].

Supplementation with human MSCs-concentrated conditioned media enhanced both cell migration and proliferation in vitro which decreased due to hypoxia and low serum VEGF concentration [31]. Human skin fibroblasts cultured with human umbilical cord blood, MSC-conditioned media, exhibited significantly elevated migratory ability [15]. Bari E et al. for the first time reported on a whole-MSC secretome formulated in a sponge-like alginate which was used for wound dressing and prepared in a public GMP-compliant facility with a scalable process. Experimental wounds in mice treated with this remedy, providing the controlled release of MSCs bioactive substances, healed faster than controls without complications or infections, and the efficacy was substantially supported by the agreement between histological and proteomic findings [33].

Human bone marrow-derived MSCs were shown to increase wound closure rate by increasing the in vitro migration of fibroblasts and keratinocytes as well as human skin fibroblasts [15]. On a model of chronic radiation cystitis in Sprague Dawley rat, intravenous injection of adipose tissue MSCs reduced vascular damage 12 months after irradiation to the same level as the non-irradiated control and reduced damage to the bladder epithelium as well as inhibited disease progression [34]. In another study, the application of mesenchymal stromal cells isolated from mouse whisker hair follicle outer root sheath in experimentally induced full-thickness wounds in syngeneic C57BL/6 mice allowed the researchers to achieve a general reduction in hypertrophic scars due to decreased inflammation, cellularity, and number of collagen filaments compared to control group [35]. The transplantation of adipose tissue-derived MSCs was shown to restore normal healing of CD18^−/−^ wounds in mice by restoring the decreased TGF-β1 concentrations; mutations in the CD18 gene encoding the common β-chain of β2 integrins is a known cause of the impaired wound healing [36]. MSCs modified with E-selectin injected into experimental ischemic wounds in mice were shown to accelerate wound healing and manifested stronger survival and viability [37].

There are just a few published clinical studies on the application of MSCs for the enhancement of wound healing; however, all of them support the safety and efficacy of non-healing wounds treatment with MSC [38,39,40,41,42]. Moreover, it should be noted that nowadays, MSCs derived products, in particular exosomes and MSC culture media, are increasingly used in clinical research [43,44].

The application of autologous bone marrow-derived cells to three patients with wounds of more than 1-year duration resulted in complete wound closure and showed evidence of dermal rebuilding. Histologic findings confirmed engraftment and reduced scarring [38]. The topical application of MSCs using a fibrin spray system was shown to be a reliable and promising approach for patients with non-healing wounds [39]. The application of artificial dermis made of collagen sponge containing composite graft consisting of cultured autologous bone marrow MSCs to twenty patients with skin wounds allowed the researchers to achieve wound healing in eighteen of them. Two patients died due to reasons unrelated to cell therapy [40].

In another study, the patients (*n* = 24) with non-healing ulcers of the lower limb were implanted with autologous-cultured bone marrow-derived MSCs. A 12-week follow-up period showed that the implant group had significant improvement in pain-free walking distance and a reduction in ulcer size as compared to those in the control group [41]. In a clinical study, 11 patients (mean age: 66.6 ± 9.5 years) with chronic venous stasis ulcers were implanted with transplanted autologous adipose tissues-derived MSCs subcutaneously around the wound and the wound bed. An improvement was observed in 75% of the ulcers, and no serious side effects were reported [42]. Besides treatment of skin injuries, MSC are increasingly used for the management of inflammatory bowel disease; the latter affects up to 40% of the population, which involved tissue damage, in particular for the healing of perianal fistulas in Crohn’s disease [45,46].

## 4. Diabetic Wounds

Diabetes affects more than 400 million people worldwide, and foot ulceration is a major complication of diabetes mellitus which results in significant human suffering and a major burden on healthcare systems [30,47]. Diabetic foot ulcer is a non-healing wound that is caused by an imbalance of various mechanisms, such as hemostasis, inflammation, immune dysregulation, collagen deposition, and angiogenesis [10,48]. A crucial role in the development of diabetic foot ulcers is played by hyperglycemia and the blockage of peripheral blood vessels [10,17].

Globally, the risk of recurrence of a diabetic foot ulcer is 50% [46], which is mediated by the reduction in proangiogenic growth factors, and consequently, as mentioned above, decreased angiogenesis [48] and decreased proliferation, migration, and functions of fibroblasts as well as an impaired inflammatory response [18]. Diabetic patients have a 25% lifetime risk of developing foot ulcers compared to those without diabetes, and it is estimated globally that one patient with diabetes undergoes a lower limb amputation every 30 s [47,48,49]. Clinic studies lack effective therapeutics targeting diabetic vascular complications, thus mesenchymal stromal cells due to their regenerative and anti-inflammatory properties have great potential in treating diabetic foot ulcer [1,49]. The use of stromal cells in diabetes mellitus is especially justified taking into account that diabetes has been shown to decrease the function of these progenitor cells, and MSCs are crucial to the wound repair process, which is hindered in diabetes mellitus [50].

In the last decade, the safety and efficiency of MSC application in the treatment of diabetic foot ulcer were demonstrated both in preclinical and clinical studies [1,16,17,48,49]. The application of MSCs to non-healing wounds in diabetic mice as shown above leads to increased angiogenesis, reduced scarring, enhanced epithelization, and granulation tissue formation [16]. The clinical efficiency of MSCs in the treatment of diabetic foot ulcers was also confirmed in experimental studies in rats [17]. In rats with streptozotocin-induced diabetes and a full-thickness foot dorsal skin wounds, the human umbilical cord mesenchymal stromal cells transplanted intravenously through the femoral vein were shown to migrate and locate to the wound tissue and helped with wound healing, partly by regulating inflammation, trans-differentiation, and providing growth factors that promote angiogenesis, cell proliferation, and collagen deposition [51].

In diabetic wound healing, both the transplantation of umbilical cord-derived MSCs and their culture medium were shown to exert beneficial effects [14]. Human umbilical cord MSCs partially restored the alterations of body weight, fasting blood glucose, serum ICAM-1 and VCAM-1 levels, the histopathology of aorta, and reversed the abnormal phosphorylation of extracellular signal-regulated kinases, and MSCs culture medium restored the altered gene expressions of *IL-6*, TNF-α, *ICAM-1*, *VCAM-1*, *BAX*, *P16*, *P53*, and *ET-1* in diabetic rats [49]. Saheli et al. also reported that MSCs culture medium affected the gene expression profiles of healing diabetic wounds in experimental studies in rats [18]. In another study, in day-14 wounds, mice treated with umbilical cord MSCs and umbilical cord MSCs culture media showed a significant difference in capillary densities and higher levels of VEGF, PDGF, and KGF expression [14]. MSCs culture medium also improved the histopathological parameters of healing diabetic wound, confirmed by wound closure percentages on day 7 [18].

Zhang J. et al. used human umbilical cord MSCs tissue sheets on poly(lactic-co-glycolic acid) (PLGA)-based scaffold for the treatment of diabetic wounds in mice. The findings suggested that human umbilical cord MSCs cultured on PLGA scaffolds improve diabetic wound healing, collagen deposition, and angiogenesis, as well as provide a novel and effective method for cell transplantation and a promising alternative for diabetic skin wound treatment [48].

The studies in modified mouse models with dermal lesions showed that allogeneic transplantation of the specific subpopulation derived from umbilical cord MSCs, the differentiated mesenchymal cells, has positive effects on wound healing by promoting local angiogenesis. The authors suggest that differentiated mesenchymal cells could play a very important role in regenerative medicine and can maybe lead to the development of a novel cell-therapeutic tool for dermal healing diseases [21].

The reported beneficial effects of MSCs in the treatment of diabetic foot ulcer were also confirmed in clinical studies [48,52,53]. Thirty patients with diabetic foot ulcers were treated with hydrogel-based allogeneic adipose-derived stromal cell sheets in a randomized, comparator-controlled, multicenter study that resulted in a higher rate of complete wound closure; Kaplan–Meier median time to complete closure was shorter than that of the control group [52]. A randomized, controlled clinical trial conducted by Qin et al. showed that the application of umbilical cord MSCs for the treatment of diabetic foot ulcers resulted in complete or gradual healing three months after cell transplantation [53].

Topical and intravenous administration of human umbilical cord mesenchymal stromal cell to 14 patients with peripheral arterial disease and incurable diabetic foot ulcer showed that ulcer disclosure was achieved for more than 95% of the lesion area for all patients within 1.5 months after treatment. The symptoms of chronic limb ischemia were alleviated, and all of the patients survived without amputation due to the recurrence of diabetic foot ulcers within 3 years after treatments [48].

## 5. Burns

Burns remain a severe public health problem; the incidence of burns requiring medical attention is nearly 11 million, making it the fourth most common injury in the world [28,54]. Based on records from the World Health Organization, globally, the number of burn-related deaths is nearly 180,000 each year [54]. Burn wound treatment is difficult and is one of the most challenging problems in the clinic [26]. Skin-specific stem cells in the tissue play a significant role in normal wound healing and skin homeostasis regulation, and their capabilities for healing burn wounds have also been demonstrated in recent studies [54]. Rangatchew et al. having analyzed three human and thirty-nine animal studies concluded that MSCs can significantly reduce inflammation, burn wound progression, and accelerate the healing rate of acute burns [28]. The effectiveness of MSCs in burn wound treatment is attributable to their regenerating and anti-inflammatory effect as well as their ability to enhance the migration of reparative cells and angiogenesis that was also confirmed in experimental studies [17]. Most studies support the hypothesis that the beneficial effects of MSCs regarding acceleration of wound healing are mediated by the secretome of these cells, i.e., their paracrine action through the release of numerous pro- and anti-inflammatory cytokines, leptin, angiogenin, and growth factors [26,28]. Human bone marrow MSCs secrete various factors, including pro- and anti-inflammatory cytokines, leptin, angiogenin, and GCSF [26].

The application of MSCs suspension or MSCs culture medium into burn wound bed of the diabetic mice resulted in a rapid wound closure which the authors explained was due to the secretion of a wide range of paracrine factors [55]. In another study, the application of culture medium of human bone marrow MSC significantly increased the wound closure area on the 15th and the 28th day after burn injury compared to the control and sham groups. The authors concluded that human bone marrow MSCs culture medium promotes skin wound healing by increasing cell proliferation, regulating collagen synthesis and collagen composition and inducing angiogenesis at the injury site. It was also shown that MSCs culture medium in burns enhances fibroblast and basal cell proliferation, collagen bundle synthesis, and vascularization, and it showed anti-inflammatory effects in the healing process of skin wounds via the activation of fibroblasts [26].

The estimation of antimicrobial and regenerative activity of the placental multipotent mesenchymal stromal cell secretome-based chitosan hydrogel (MSC-Ch-gel) in *Staphylococcus aureus*-infected third-degree burn wounds in rats showed that it effectively promoted infected wound healing in rats with third-degree burns. Gel preparation also cleared the wound of microorganisms (*S. aureus* was not detected in the washings from the burned areas) and was shown to possess anti-inflammatory effect and enhance re-epithelialization and the growth of well-vascularized granulation tissue [56].

In another study, wound healing was significantly accelerated in the human umbilical cord MSCs therapy group. MSCs were shown to migrate into wound and decrease the number of infiltrated inflammatory cells as well as the level of IL-1, IL-6, and TNF-α and increased the levels of IL-10 and TSG-6 in wounds. The wounds treated with umbilical cord MSCs were also characterized by enhanced neovascularization and VEGF level as well as the ratio of collagen types I and III [57].

A single application of allogeneic MSCs was shown to improve the rate of burn wound healing and the histological appearance of the burn wound. MSC-treated wounds showed increased collagen content, increased epidermal area, and increased dermal thickness compared to wounds treated with culture media monocytes [58]. Several studies have also explored the therapeutic potential of stem/progenitor cells for radiation burns treatment and showed their beneficial effects [1].

## 6. Non-Union Fractures

The incidence of non-unions can be as high as 20% for certain injuries [12,59]. To date, most common procedures for the enhancement of bone regeneration rely on autologous or allogeneic bone grafts; however, the latter is characterized by several limitations, namely the risk of immunologic rejection and infection [60]. Similar to wound healing, the healing of bone fracture is a complex process in which a crucial role belongs to progenitor cells, recruited to the fracture site in the initial stages of healing (day one) and proliferate at around day three [11,60]. Impaired fracture healing in diabetes may be caused by reduced MSCs proliferation and increased MSCs apoptosis that are enhanced by TNFα as shown in mice with streptozotocin-induced diabetes [61]. MSCs, due to their ability to differentiate into osteocytes and chondrocytes along with prominent anti-inflammatory properties, are widely applied for the enhancement of fracture healing and bone bioengineering [11,12,13,29]. The local transplantation of MSCs in poorly healing fractures was shown to support bone regeneration [62].

In a bone fracture model, endogenous bone marrow resident, CD73-EGFP^+^, MSCs were found to migrate to the fracture site and differentiate into cartilage and bone cells [13]. In orthopedics and traumatology, mesenchymal stomal cells are applied both as a monotherapy and combined with growth factors [12,63]. Scaffolds seeded with MSCs are most often used in tissue engineering and include biotic and abiotic materials [11]. Bone marrow-derived MSCs transplant along with systemic subcutaneous delivery of recombinant insulin-like growth factor (IGF)-I were reported to increase the fracture callus volume in mice with a stabilized tibia fracture compared with untreated animals [63]. Another study investigating the joint effects of using MSC sheets with local injection of stromal cell-derived factor-1 (SDF-1) on bone formation showed complete bone union at 8 weeks, whereas the control group showed non-union of the bone [12]. In experimental studies in dogs with transverse radial fractures at the radius, MSCs sheets were shown to reduce the quantity of external callus, and osteogenic differentiated mesenchymal stromal cells induced the primary bone healing [28].

Clinically, MSC’s application was shown to be characterized by a significantly reduced incidence of poor recovery, and patients with bone fractures were reported to benefit from MSCs administration although larger randomized clinical trials are necessary to confirm these findings [64]. The preservation of endogenous MSCs namely through the decrease in negative effect of TNFα on this cell population can maximize regenerative potential in diabetic patients with bone fractures [61].

## 7. Pressure Ulcers

Pressure ulcers are lesions of the skin and underlying tissues placed under continuous pressure [65,66]. Nowadays, pressure ulcers are increasing worldwide with the aging population; however, there is no effective treatment for this type of wounds [65]. Since MSCs were reported to accelerate the healing of wounds of different origin (traumatic, diabetic, venous ulcers, and burns), some research groups made attempts to utilize the potential of cell-based therapies in pressure ulcers. According to our knowledge, most papers evaluating the potential of MSC to treat pressure ulcers originate from preclinical research [65,67,68,69]. Sario GD et al., based on the analysis of scientific articles on animal models using MSCs as primary therapy for pressure ulcers and published between 2008 and 2020, concluded that MSCs have shown promising results in treating this type of wounds both in animal and in clinical trials [70].

Having created an immortalized stem cell line from human-exfoliated deciduous teeth, Yasuhiro Katahira et al. investigated the effects of the conditioned medium of MSCs on cutaneous pressure ulcers formation induced by ischemia–reperfusion injury. For the first time, these authors showed that an immortalized stem cell line from human-exfoliated deciduous teeth exerts was beneficial in treating pressure ulcers formation by stimulating angiogenesis and increasing oxidative stress resistance through vascular endothelial growth factor and hepatocyte growth factor [65]. In another study, pressure ulcers were induced via the application of magnetic devices on the dorsal skin of immunodeficient NOD/SCID mice, which causes localized ischemia. The model may be applicable for experimental pressure ulcer research; however, intradermal transplantation of human MSCs did not accelerate healing in this study [67]. The study investigating the effects of adipose-derived MSCs from type 2 diabetes mellitus patients on wound healing of pressure ulcers in mice found that MSC therapy resulted in the acceleration of wound healing by promoting angiogenesis, collagen deposition, and Schwann cell regeneration [68]. Another study reported the effective induction of pressure ulcer healing by MSC secretome in C57Bl/6 mice [69]. The safety and efficacy of human adipose tissue stem cells were confirmed also in the study by Joanna Bukowska et al. on a murine skin pressure ulcer model where dermal fibroblast cells were used as a control. Both fresh and cryopreserved human adipose tissue stem cells significantly accelerated the processes of wound healing in young mice of both sexes relative to the dermal fibroblast cells in the controls [66].

## 8. Lower Extremity Venous Ulcers

Venous ulcers of the lower extremities are serious and recurrent complications of the chronic venous insufficiency of the lower limbs negatively affect patients’ daily life [71]. In the last decade, MSC-based therapies are increasingly investigated for the management of venous ulcers of the lower extremities [39,42,71,72,73]. A systemic review conducted by Elsharkawi M. et al. on the safety and effectiveness of adipose-derived stem cells in 66 patients with venous lower extremities ulcers showed that stem cell therapy facilitates wound healing in chronic venous insufficiency of the lower extremities along with a decrease in pain scores [72]. Another pilot study reports treatment outcomes in 11 patients with chronic venous stasis ulcers with autologous adipose tissue-derived MSCs. A cell concentrate was implanted subcutaneously around the wound and the wound bed in addition to standard local and general treatment. The authors reported no serious adverse events and observed an improvement in 75% of the ulcers, supposing this cell-based approach to be safe and promising [39,42]. Bone marrow MSCs were shown to accelerate the healing rate of the lower extremities of venous ulcers by about 10-fold compared to those in the saline and fibrin control groups in a small (*n* = 11 patients) randomized, controlled, and double-blinded pilot clinical trial [39]. In the interventional, multicenter, single-arm, phase I/IIa clinical trial, 31 patients (16 male and 15 female) with therapy-resistant chronic venous ulcers received one or two topical applications of 1 × 10^6^ skin-derived allogeneic ABCB5^+^ mesenchymal stem cells per cm^2^ wound area in addition to conventional treatment. Wound area at week 12 decreased by 76% [73]. Jiao L et al. also reported a clinical case of a patient with a lower extremity venous ulcer who had not healed for a period of up to 1 year. The injection of the human umbilical MSCs and platelet-rich plasma into the wound edge and base resulted in a decrease in the wound area by nearly 50%; the ulcer had almost completely healed by day 62 with no serious adverse effects [71].

## 9. Epidermolysis Bullosa

Epidermolysis bullosa is a group of incurable, inherited mucocutaneous fragility disorders, characterized by the formation of severe, chronic blisters with painful and life-threatening complications [74,75]. Emerging treatments for epidermolysis bullosa like gene therapy, bone marrow transplantation, cell therapy (allogenic fibroblasts, MSCs), tissue-engineered, and skin-like structures are promising approaches for the management of this disease [74]. Intradermal and intravenous injections of mesenchymal stromal cells have shown therapeutic potential for epidermolysis bullosa patients [75,76,77,78].

In experimental studies, the intradermal administration of MSCs in an epidermolysis bullosa mouse model resulted in the production and deposition of C7 at the dermal–epidermal junction, the physiological site of function. Additional benefits were gained from MSCs’ anti-inflammatory effects, which led to decreased immune cell infiltration into the injured epidermolysis bullosa skin and promoted the regeneration of epidermolysis bullosa wounds [79].

In 2021, the outcomes of the first clinical trial of systemic administration of umbilical cord blood-derived MSCs in patients (four adult and two pediatric patients) with epidermolysis bullosa were published, demonstrating the safety and the transient clinical benefits of this therapy. The intravenous administration of umbilical cord blood-derived MSCs in epidermolysis bullosa was well tolerated; improvements in the Birmingham epidermolysis bullosa severity score, body surface area involvement, blister counts, pain, pruritus, and quality of life were observed with maximal effects at 56–112 days after treatment [77]. In an earlier study involving 10 epidermolysis bullosa in adults, the intravenous infusions of bone marrow MSCs (twice) were well tolerated with a significant reduction in itch and a transient decrease in disease activity score (8/10 patients). One patient showed a transient increase in type VII collagen [78].

In 2020, Maseda et al. published the first report on the use of systemic allogeneic adipose tissue-derived MSC in a patient with epidermolysis bullosa harboring heterozygous biallelic *COL7A1* gene mutations along with a diminished expression of C7. The authors noted improvements in wound healing, itch, pain, and quality of life, maximally 6–9 months after cell therapy with a noticeable relief of symptoms of up to 2 years [75].

The good tolerability, manageable safety, and potential efficacy of intravenous infusions of dermal ABCB5^+^ MSCs in epidermolysis bullosa were shown in the international, multicentric, single-arm, phase I/IIa clinical trial that enrolled 16 patients (aged 4–36 years). The patients received three intravenous infusions of 2 × 10^6^ ABCB5^+^ MSCs/kg. The patients showed improvements according to several clinical assessment scores as well as reductions in itch and pain numerical rating scale scores during the 12 weeks follow-up [80].

## 10. Advantages and Limitations of MSC’s Application in Wound Healing

Numerous preclinical and clinical studies reveal the benefits of MSC’s application for the facilitation of chronic wounds healing. The unique advantage of this biological approach is the opportunity to modulate natural wound healing environment by affecting all stages of the wound healing process in particular through the regenerative, anti-inflammatory, and immunomodulatory effect of MSCs [3,11,12,14,15,16]. The stimulation of neovasculogenesis, extracellular matrix deposition, and bactericidal effect, reported in studies in vitro, as well as the ability of MSCs to differentiate into the cells of various lineages including skin cells makes them especially promising for the treatment of injuries of different origin [1,5,9,10,11,12,16,18,22]. However, MCS therapy nowadays has some limitations.

First of all, clinical trials exploring MSCs in healing chronic wounds mostly enrolled small number of patients and used different methodology, thus large-scale clinical trials with standardized evaluation approach are needed [1]. The controversial occurrence of malignant transformation of MSCs raises concerns about the safety of cell therapies; however, protocols that allow for the accurate verification of the quality and safety of MSCs are being developed [81]. Recent papers stress the importance for the standardization of the processes of manufacturing and testing of MSC-based advanced tissue medicinal products [82].

Another important controversial issue in MSC-based therapy of wounds is the optimal delivery method. The methods of MSC delivery described in clinical trials on wounds therapy involve local injection/transplantation of MSC (sometimes endoscopy or ultrasonography guided), direct topical application on wound bed, intrauterine injection, intravenous infusion, percutaneous injection, and others. The choice of the optimal MSC carrier is also disputable. Different gel carriers, hydrogel sheets, fibrin glues, scaffolds, matrix, and skin grafts were used in clinical trials (Table 1 and Table 2) [1,15,30,83]. Scaffolds, matrix, and hydrogels provide a good environment for tissue regeneration, proliferation, and the differentiation of the cells. Optionally, the use of such compounds such as glycol, sodium alginate/collagen hydrogel, chitosan, peptide, timolol, and poly(vinyl) alcohol along with cell-based therapy is considered to have beneficial effects in wound management [83]. Extracellular matrix constituents provide the tissue with three-dimensional (3D) structural integrity, and cellular-function regulation prompts stem-cell proliferation and differentiation for 3D organoid construction in vitro or tissue regeneration in vivo [84]. Further investigations and large-scale clinical trials are necessary for the validation of the most suitable MSC delivery routes and carriers.

## 11. Summary and Future Perspectives

Chronic wounds remain a persistent challenge in healthcare due to their high prevalence and limited effective treatments. Mesenchymal stromal cells (MSCs), with their regenerative, anti-inflammatory, and immunomodulatory properties, are promising candidates for improving wound healing, particularly in chronic wounds, diabetic ulcers, burns, non-union fractures, and genetic disorders like epidermolysis bullosa. MSCs enhance healing primarily through paracrine signaling, releasing trophic factors that stimulate cellular proliferation, angiogenesis, and immune modulation. Their low immunogenicity makes MSCs suitable for both autologous and allogeneic therapies. Research demonstrates that MSCs aid in every stage of wound healing; they reduce inflammation, activate fibroblast activity, and promote new capillary formation. The clinical applications of MSCs, including cell-free MSC secretome products, have shown safety and efficacy in accelerating wound closure, reducing scarring, and improving outcomes in challenging wound types. Future advancements should focus on optimizing MSC delivery and engraftment through innovative vehicles like scaffolds and hydrogels, expanding the use of cell-free therapies such as MSC-derived exosomes and refining applications to suit specific wound types, especially in diabetic ulcers and in genetic skin conditions, through 3D bioprinting and tissue engineering. Moving forward, large-scale, randomized clinical trials are essential to establish standardized protocols, optimal dosages, and delivery routes, furthering MSC-based therapies as transformative solutions in wound care.

## Figures and Tables

**Table 1:** Recruiting clinical trials applying MSCs for the enhancement of tissues healing.

Condition	Method of Cell Delivery	Combination Agent	Autologous/ Allogeneic	Cell Source	Phase	NCT Number
Radiation-induced rectal injury	Local application by means of endoscopy	-	Allogeneic	Umbilical cord	1	05939778
Anterior cruciate ligament reconstruction using mesenchymal stem cells and collagen matrix carrier	Local implantation	Porous bovine collagen matrix carrier	Autologous	Anterior cruciate ligament	N/A	05582226
Venous leg	Local transplantation	Silver ion dressing	Allogeneic	Umbilical cord	N/A	05319106
Dystrophic epidermolysis bullosa	Local application of MSC dressing	Dressing for dystrophic epidermolysis bullosa wound	Allogeneic	N/A	2	05157958
Skin grafts in donor site wounds	MSC injection into the de-epithelialized area and the surrounding 0.5 cm subcutaneous region.	-	Allogeneic	Umbilical cord	N/A	05984628

**Table 2:** Completed clinical trials applying MSCs for the enhancement of tissues healing.

Condition	Method of Cell Delivery	Combination Agent	Autologous/ Allogeneic	Cell Source	Phase	NCT Number
Mandible fractures	Local application on the fracture site within the surgical procedure	-	Autologous	Adipose tissue	3	02755922
Tibial closed diaphyseal fractures	Local application in the fractured site	-	Allogeneic	Adipose tissue	2	02140528
Tendon injury	Ultrasound-guided injection at the injury site	-	Allogeneic	Adipose tissue	1	01856140
Heel injury	Local application	Skin graft	Allogeneic	Umbilical cord	1	NCT04219657
Poor healing after uterus injury	Intrauterine injection	-	Allogeneic	Umbilical cord	1	03386708
Second degree burn wounds of less than 20% of the total body surface area	Local application	-	Allogeneic	N/A	1	02104713
Chronic wounds in diabetic foot syndrome	Direct application onto the prepared wound bed	Fibrin gel	Allogeneic	Adipose tissue	1–2	03865394
Non-union of long bone fractures	Local application in fractured zone	-	Autologous	Bone marrow	1	01206179
Non-united tibial and femoral fractures	Injection in non-union site	-	N/A	Bone marrow	2	01788059
Tendon injury	Local injection under ultrasound guidance	Fibrin glue + range of motion exercise	Allogeneic	Adipose tissue	2	02298023
Distal tibial fractures	Local implantation at the fracture site	MSC carrier	Autologous	N/A	1–2	00250302
Ocular corneal burn	Subconjunctival injection	-	N/A	Bone marrow	2	02325843
Fracture non-union healing	Local application of in vitro-expanded MSC	Carrier	Autologous	Bone marrow	N/A	02177565
Chronic ulcer wounds	Topical application of Wharton jelly MSC culture medium	Gel carrier	Allogeneic	Umbilical cord	1	04134676
Knee articular cartilage injury	Local application	-	Allogeneic	Umbilical cord	3	01041001
Deep second-degree burn wound	Local application	hydrogel sheet	Allogeneic	Adipose tissue	1	02394873
Long bones non-union	Percutaneous application around fracture ends	-	Autologous	Adipose tissue	1–2	04340284
Knee articular cartilage injury or defect	Local application	-	Allogeneic	Umbilical cord	3	01626677
Support of autologous chondron transplantation with instant MSC	Filling of cartilage defect	Glue carrier, autologous chondrons	Allogeneic	N/A	1–2	02037204
Tibial shaft fracture	Local injection	-	Autologous	Bone marrow	N/A	00512434
Mandibular distraction osteogenesis	Local injection	-	N/A	Bone marrow	N/A	03861650
Severe epidermolysis bullosa	Serial infusions	Allogeneic hematopoietic stem cell transplant	Allogeneic (related)	N/A	2	02582775
Severe epidermolysis bullosa	Infusions	Allogeneic hematopoietic stem cell transplant	Allogeneic	N/A	1–2	01033552
Recessive dystrophic epidermolysis bullosa	Infusions	-	Allogeneic	Umbilical cord blood	1–2	04520022
Epidermolysis bullosa	Local application	hydrogel sheet	Allogeneic	Adipose tissue	1–2	02579369
Epidermolysis bullosa	Local application	hydrogel sheet	Allogeneic	Adipose tissue	1–2	03183934
Treatment-refractory chronic venous ulcers	Topical application	-	Allogeneic	Skin derived	1–2	03257098
Recessive dystrophic epidermolysis bullosa	Infusions	-	Allogeneic	Skin derived	1–2A	03529877

## References

[1] Huang Y.-Z., Gou M., Da L.-C., Zhang W.-Q., Xie H.-Q. (2020). Mesenchymal stem cells for chronic wound healing: Current status of preclinical and clinical studies. Tissue Eng. Part B Rev..

[2] Mirhaj M., Labbaf S., Tavakoli M., Seifalian A.M. (2022). Emerging treatment strategies in wound care. Int. Wound J..

[3] Isakson M., De Blacam C., Whelan D., McArdle A., Clover A.J.P. (2015). Mesenchymal stem cells and cutaneous wound healing: Current evidence and future potential. Stem Cells Int..

[4] Ong H.T., Dilley R.J. (2018). Novel non-angiogenic role for mesenchymal stem cell-derived vascular endothelial growth factor on keratinocytes during wound healing. Cytokine Growth Factor Rev..

[5] Millán-Rivero J.E., Martínez C.M., Romecín P.A., Aznar-Cervantes S.D., Carpes-Ruiz M., Cenis J.L., Moraleda J.M., Atucha N.M., García-Bernal D. (2019). Silk fibroin scaffolds seeded with Wharton’s jelly mesenchymal stem cells enhance re-epithelialization and reduce formation of scar tissue after cutaneous wound healing. Stem Cell Res. Ther..

[6] Wu S., Sun S., Fu W., Yang Z., Yao H., Zhang Z. (2024). The Role and Prospects of Mesenchymal Stem Cells in Skin Repair and Regeneration. Biomedicines.

[7] Sopata M., Jawień A., Mrozikiewicz-Rakowska B., Augusewicz Z., Bakowska M., Samson I., Gabriel M., Grzela T., Karpiński T., Kuberka I. (2020). Wytyczne postępowania miejscowego w ranach niezakażonych, zagrożonych infekcją oraz zakażonych—Przegląd dostępnych substancji przeciwdrobnoustrojowych stosowanych w leczeniu ran. Zalecenia Polskiego Towarzystwa Leczenia Ran. Leczenie Ran.

[8] Maxson S., Lopez E.A., Yoo D., Danilkovitch-Miagkova A., LeRoux M.A. (2012). Concise Review: Role of Mesenchymal stem cells in wound Repair. Stem Cells Transl. Med..

[9] Cappuzzello C., Doni A., Dander E., Pasqualini F., Nebuloni M., Bottazzi B., Mantovani A., Biondi A., Garlanda C., D’Amico G. (2016). Mesenchymal stromal Cell-Derived PTX3 promotes wound healing via fibrin remodeling. J. Investig. Dermatol..

[10] Gofron M., Mrozikiewicz-Rakowska B., Sieńko D., Czupryniak L. (2021). Możliwości zwiększenia efektywności terapii leczenia ran za pomocą mezenchymalnych komórek macierzystych u osób chorych na cukrzycę. Diabetol. Prakt..

[11] Ramos-Gonzalez G., Salazar L., Wittig O., Diaz-Solano D., Cardier J.E. (2022). The effects of mesenchymal stromal cells and platelet-rich plasma treatments on cutaneous wound healing. Arch. Dermatol. Res..

[12] Chen G., Fang T., Qi Y., Yin X., Di T., Feng G., Lei Z., Zhang Y., Huang Z. (2016). Combined use of mesenchymal stromal cell sheet transplantation and local injection of SDF-1 for bone repair in a rat nonunion model. Cell Transplant..

[13] Kimura K., Breitbach M., Schildberg F.A., Hesse M., Fleischmann B.K. (2021). Bone marrow CD73+ mesenchymal stem cells display increased stemness in vitro and promote fracture healing in vivo. Bone Rep..

[14] Shrestha C., Zhao L., Chen K., He H., Mo Z. (2013). Enhanced healing of diabetic wounds by subcutaneous administration of human umbilical cord derived stem cells and their conditioned media. Int. J. Endocrinol..

[15] Lee D.E., Ayoub N., Agrawal D.K. (2016). Mesenchymal stem cells and cutaneous wound healing: Novel methods to increase cell delivery and therapeutic efficacy. Stem Cell Res. Ther..

[16] Shabbir A., Cox A., Rodriguez-Menocal L., Salgado M., Van Badiavas E. (2015). Mesenchymal stem cell exosomes induce proliferation and migration of normal and chronic wound fibroblasts, and enhance angiogenesis in vitro. Stem Cells Dev..

[17] Guillamat-Prats R. (2021). The role of MSC in wound healing, scarring and regeneration. Cells.

[18] Saheli M., Bayat M., Ganji R., Hendudari F., Kheirjou R., Pakzad M., Najar B., Piryaei A. (2019). Human mesenchymal stem cells-conditioned medium improves diabetic wound healing mainly through modulating fibroblast behaviors. Arch. Dermatol. Res..

[19] Jackson W.M., Nesti L.J., Tuan R.S. (2011). Concise Review: Clinical Translation of wound healing therapies based on mesenchymal stem cells. Stem Cells Transl. Med..

[20] An Y., Liu W.J., Xue P., Ma Y., Zhang L.Q., Zhu B., Qi M., Li L.Y., Zhang Y.J., Wang Q.T. (2018). Autophagy promotes MSC-mediated vascularization in cutaneous wound healing via regulation of VEGF secretion. Cell Death Dis..

[21] Palma M.B., Luzzani C., Andrini L.B., Riccillo F., Buero G., Pelinski P., Inda A.M., Errecalde A.L., Miriuka S., Carosella E.D. (2021). Wound healing by allogeneic transplantation of specific subpopulation from human umbilical cord mesenchymal stem cells. Cell Transplant..

[22] Chow L., Johnson V., Impastato R., Coy J., Strumpf A., Dow S. (2019). Antibacterial activity of human mesenchymal stem cells mediated directly by constitutively secreted factors and indirectly by activation of innate immune effector cells. Stem Cells Transl. Med..

[23] Rodriguez-Menocal L., Shareef S., Salgado M., Shabbir A., Van Badiavas E. (2015). Role of whole bone marrow, whole bone marrow cultured cells, and mesenchymal stem cells in chronic wound healing. Stem Cell Res. Ther..

[24] Zhang J., Guan J., Niu X., Hu G., Guo S., Li Q., Xie Z., Zhang C., Wang Y. (2015). Exosomes released from human induced pluripotent stem cells-derived MSCs facilitate cutaneous wound healing by promoting collagen synthesis and angiogenesis. J. Transl. Med..

[25] Jiang Z., Liu G., Meng F., Wang W., Hao P., Xiang Y., Wang Y., Han R., Li F., Wang L. (2017). Paracrine effects of mesenchymal stem cells on the activation of keratocytes. Br. J. Ophthalmol..

[26] Aryan A., Bayat M., Bonakdar S., Taheri S., Haghparast N., Bagheri M., Piryaei A., Abdollahifar M.-A. (2018). Human bone marrow mesenchymal stem cell conditioned medium promotes wound healing in deep Second-Degree burns in male rats. Cells Tissues Organs.

[27] Yao B., Huang S., Gao D., Xie J., Liu N., Fu X. (2015). Age-associated changes in regenerative capabilities of mesenchymal stem cell: Impact on chronic wounds repair. Int. Wound J..

[28] Rangatchew F., Vester-Glowinski P., Rasmussen B.S., Haastrup E., Munthe-Fog L., Talman M.-L., Bonde C., Drzewiecki K.T., Fischer-Nielsen A., Holmgaard R. (2021). Mesenchymal stem cell therapy of acute thermal burns: A systematic review of the effect on inflammation and wound healing. Burns.

[29] Yoon B.S., Moon J.-H., Jun E.K., Kim J., Maeng I., Kim J.S., Lee J.H., Baik C.S., Kim A., Cho K.S. (2010). Secretory profiles and wound healing effects of human amniotic Fluid–Derived mesenchymal stem cells. Stem Cells Dev..

[30] Du S., Zeugolis D.I., O’Brien T. (2022). Scaffold-based delivery of mesenchymal stromal cells to diabetic wounds. Stem Cell Res. Ther..

[31] Kosol W., Kumar S., Marrero-BerrÍos I., Berthiaume F. (2020). Medium conditioned by human mesenchymal stromal cells reverses low serum and hypoxia-induced inhibition of wound closure. Biochem. Biophys. Res. Commun..

[32] Malhotra P., Shukla M., Meena P., Kakkar A., Khatri N., Nagar R.K., Kumar M., Saraswat S.K., Shrivastava S., Datt R. (2021). Mesenchymal stem cells are prospective novel off-the-shelf wound management tools. Drug Deliv. Transl. Res..

[33] Bari E., Di Silvestre D., Mastracci L., Grillo F., Grisoli P., Marrubini G., Nardini M., Mastrogiacomo M., Sorlini M., Rossi R. (2020). GMP-compliant sponge-like dressing containing MSC lyo-secretome: Proteomic network of healing in a murine wound model. Eur. J. Pharm. Biopharm..

[34] Brossard C., Pouliet A.-L., Lefranc A., Benadjaoud M., Santos M.D., Demarquay C., Buard V., Benderitter M., Simon J.-M., Milliat F. (2023). Mesenchymal stem cells limit vascular and epithelial damage and restore the impermeability of the urothelium in chronic radiation cystitis. Stem Cell Res. Ther..

[35] Li H., Ziemer M., Stojanovic I., Saksida T., Maksimovic-Ivanic D., Mijatovic S., Djmura G., Gajic D., Koprivica I., Krajnovic T. (2022). Mesenchymal stem cells from mouse hair follicles reduce hypertrophic scarring in a murine wound healing model. Stem Cell Rev. Rep..

[36] Jiang D., Scharffetter-Kochanek K. (2020). Mesenchymal stem cells adaptively respond to environmental cues thereby improving granulation tissue formation and wound healing. Front. Cell Dev. Biol..

[37] Huerta C.T., Ortiz Y.Y., Li Y., Ribieras A.J., Voza F., Le N., Dodson C., Wang G., Vazquez-Padron R.I., Liu Z.-J. (2023). Novel Gene-Modified Mesenchymal stem cell therapy reverses impaired wound healing in ischemic limbs. Ann. Surg..

[38] Badiavas E.V. (2003). Treatment of chronic wounds with Bone Marrow–Derived cells. Arch. Dermatol..

[39] Falanga V., Iwamoto S., Chartier M., Yufit T., Butmarc J., Kouttab N., Shrayer D., Carson P. (2007). Autologous bone Marrow–Derived cultured mesenchymal stem cells delivered in a fibrin spray accelerate healing in murine and human cutaneous wounds. Tissue Eng..

[40] Yoshikawa T., Mitsuno H., Nonaka I., Sen Y., Kawanishi K., Inada Y., Takakura Y., Okuchi K., Nonomura A. (2008). Wound therapy by marrow mesenchymal cell transplantation. Plast. Reconstr. Surg..

[41] Dash S.N., Dash N.R., Guru B., Mohapatra P.C. (2013). Towards reaching the target: Clinical application of mesenchymal stem cells for diabetic foot ulcers. Rejuvenation Res..

[42] Masłowski L., Paprocka M., Czyżewska-Buczyńska A., Bielawska-Pohl A., Duś D., Grendziak R., Witkiewicz W., Czarnecka A. (2020). Autotransplantation of the adipose Tissue-Derived mesenchymal stromal cells in therapy of venous stasis ulcers. Arch. Immunol. Et Ther. Exp..

[43] Hade M.D., Suire C.N., Mossell J., Suo Z. (2022). Extracellular vesicles: Emerging frontiers in wound healing. Med. Res. Rev..

[44] Bian D., Wu Y., Song G., Azizi R., Zamani A. (2022). The application of mesenchymal stromal cells (MSCs) and their derivative exosome in skin wound healing: A comprehensive review. Stem Cell Res. Ther..

[45] Eiro N., Fraile M., González-Jubete A., González L.O., Vizoso F.J. (2022). Mesenchymal (STEM) stromal cells based as new therapeutic alternative in inflammatory bowel disease: Basic mechanisms, experimental and clinical evidence, and challenges. Int. J. Mol. Sci..

[46] Carvello M., Lightner A., Yamamoto T., Kotze P.G., Spinelli A. (2019). Mesenchymal stem cells for perianal Crohn’s disease. Cells.

[47] Jodheea-Jutton A., Hindocha S., Bhaw-Luximon A. (2022). Health economics of diabetic foot ulcer and recent trends to accelerate treatment. Foot.

[48] Zhang C., Huang L., Wang X., Zhou X., Zhang X., Li L., Wu J., Kou M., Cai C., Lian Q. (2022). Topical and intravenous administration of human umbilical cord mesenchymal stem cells in patients with diabetic foot ulcer and peripheral arterial disease: A phase I pilot study with a 3-year follow-up. Stem Cell Res. Ther..

[49] Liu Y., Chen J., Liang H., Cai Y., Li X., Yan L., Zhou L., Shan L., Wang H. (2022). Human umbilical cord-derived mesenchymal stem cells not only ameliorate blood glucose but also protect vascular endothelium from diabetic damage through a paracrine mechanism mediated by MAPK/ERK signaling. Stem Cell Res. Ther..

[50] Zhuge Y., Regueiro M.M., Tian R., Li Y., Xia X., Vazquez-Padron R., Elliot S., Thaller S.R., Liu Z.-J., Velazquez O.C. (2018). The effect of estrogen on diabetic wound healing is mediated through increasing the function of various bone marrow-derived progenitor cells. J. Vasc. Surg..

[51] Shi R., Lian W., Jin Y., Cao C., Han S., Yang X., Zhao S., Li M., Zhao H. (2020). Role and effect of vein-transplanted human umbilical cord mesenchymal stem cells in the repair of diabetic foot ulcers in rats. Acta Biochim. Et Biophys. Sin..

[52] Moon K.-C., Suh H.-S., Kim K.-B., Han S.-K., Young K.-W., Lee J.-W., Kim M.-H. (2019). Potential of Allogeneic Adipose-Derived Stem Cell–Hydrogel complex for treating diabetic foot ulcers. Diabetes.

[53] Qin H., Zhu X., Zhang B., Zhou L., Wang W. (2016). Clinical evaluation of human umbilical cord mesenchymal stem cell transplantation after angioplasty for diabetic foot. Exp. Clin. Endocrinol. Diabetes.

[54] Wang M., Xu X., Lei X., Tan J., Xie H. (2021). Mesenchymal stem cell-based therapy for burn wound healing. Burn. Trauma.

[55] Lykov A.P., Bondarenko N.A., Poveshchenko O.V., Miller T.V., Poveshchenko A.F., Surovtseva M.A., Bgatova N.P., Konenkov V.I. (2016). Biomedical cellular product for wound healing. Integr. Obes. Diabetes.

[56] Kudinov V.A., Artyushev R.I., Zurina I.M., Lapshin R.D., Snopova L.B., Mukhina I.V., Grinakovskaya O.S., Saburina I.N. (2021). Antimicrobial and regenerative effects of placental multipotent mesenchymal stromal cell Secretome-Based Chitosan gel on infected burns in rats. Pharmaceuticals.

[57] Liu X., Wu M., Peng Y., Chen X., Sun J., Huang F., Fan Z., Zhou H., Wu X., Yu G. (2013). Improvement in Poor Graft Function after Allogeneic Hematopoietic Stem Cell Transplantation upon Administration of Mesenchymal Stem Cells from Third-Party Donors: A Pilot Prospective Study. Cell Transplant..

[58] Clover A.J.P., Kumar A.H.S., Isakson M., Whelan D., Stocca A., Gleeson B.M., Caplice N.M. (2015). Allogeneic mesenchymal stem cells, but not culture modified monocytes, improve burn wound healing. Burns.

[59] Nicholson J., Makaram N., Simpson A., Keating J. (2021). Fracture nonunion in long bones: A literature review of risk factors and surgical management. Injury.

[60] Carvalho M.S., Poundarik A.A., Cabral J.M.S., Da Silva C.L., Vashishth D. (2018). Biomimetic matrices for rapidly forming mineralized bone tissue based on stem cell-mediated osteogenesis. Sci. Rep..

[61] Ko K.I., Coimbra L.S., Tian C., Alblowi J., Kayal R.A., Einhorn T.A., Gerstenfeld L.C., Pignolo R.J., Graves D.T. (2015). Diabetes reduces mesenchymal stem cells in fracture healing through a TNFα-mediated mechanism. Diabetologia.

[62] Rapp A.E., Bindl R., Heilmann A., Erbacher A., Müller I., Brenner R.E., Ignatius A. (2015). Systemic mesenchymal stem cell administration enhances bone formation in fracture repair but not load-induced bone formation. Eur. Cells Mater..

[63] Myers T.J., Yan Y., Granero-Molto F., Weis J.A., Longobardi L., Li T., Li Y., Contaldo C., Ozkhan H., Spagnoli A. (2012). Systemically delivered insulin-like growth factor-I enhances mesenchymal stem cell-dependent fracture healing. Growth Factors.

[64] Yi H., Wang Y., Liang Q., Mao X. (2022). Preclinical and Clinical Amelioration of Bone Fractures with Mesenchymal Stromal Cells: A Systematic Review and Meta-Analysis. Cell Transplant..

[65] Katahira Y., Murakami F., Inoue S., Miyakawa S., Sakamoto E., Furusaka Y., Watanabe A., Sekine A., Kuroda M., Hasegawa H. (2023). Protective effects of conditioned media of immortalized stem cells from human exfoliated deciduous teeth on pressure ulcer formation. Front. Immunol..

[66] Bukowska J., Alarcon Uquillas A., Wu X., Frazier T., Walendzik K., Vanek M., Gaupp D., Bunnell B.A., Kosnik P., Mehrara B. (2020). Safety and Efficacy of Human Adipose-Derived Stromal/Stem Cell Therapy in an Immunocompetent Murine Pressure Ulcer Model. Stem Cells Dev..

[67] de la Garza-Rodea A.S., Knaän-Shanzer S., van Bekkum D.W. (2011). Pressure ulcers: Description of a new model and use of mesenchymal stem cells for repair. Dermatology.

[68] Deng C.L., Yao Y.Z., Liu Z.Y., Wang B., Wang D.L., Wei Z.R. (2019). Effects of adipose-derived mesenchymal stem cells from type 2 diabetes mellitus patients on wound healing of pressure ulcers in mice. Zhonghua Shao Shang Za Zhi.

[69] Alexandrushkina N., Nimiritsky P., Eremichev R., Popov V., Arbatskiy M., Danilova N., Malkov P., Akopyan Z., Tkachuk V., Makarevich P. (2020). Cell Sheets from Adipose Tissue MSC Induce Healing of Pressure Ulcer and Prevent Fibrosis via Trigger Effects on Granulation Tissue Growth and Vascularization. Int. J. Mol. Sci..

[70] Sario G.D., Borna S., Eldaly A.S., Quinones-Hinojosa A., Zubair A.C., Ho O.A., Forte A.J. (2023). Mesenchymal Stromal Cell Healing Outcomes in Clinical and Pre-Clinical Models to Treat Pressure Ulcers: A Systematic Review. J. Clin. Med..

[71] Jiao L., Nie J., Duan L., Qiao X., Sui Y. (2024). Umbilical cord mesenchymal stem cells combined with autologous platelet-rich plasma for lower extremity venous ulcers: A case report and literature review. Medicine.

[72] Elsharkawi M., Ghoneim B., Westby D., Jones D., Tawfick W., Walsh S.R. (2023). Adipose-derived stem cells in patients with venous ulcers. Syst. Rev. Vasc..

[73] Kerstan A., Dieter K., Niebergall-Roth E., Dachtler A.K., Kraft K., Stücker M., Daeschlein G., Jünger M., Görge T., Meyer-Pannwitt U. (2022). Allogeneic ABCB5^+^ mesenchymal stem cells for treatment-refractory chronic venous ulcers: A phase I/IIa clinical trial. JID Innov..

[74] Nita M., Pliszczyński J., Eljaszewicz A., Moniuszko M., Ołdak T., Woźniak K., Majewski S., Kowalewski C., Kamiński A., Śladowski D. (2021). Surgical treatment of wounds using stem cells in epidermolysis Bullosa (EB). IntechOpen eBooks.

[75] Maseda R., Martínez-Santamaría L., Sacedón R., Butta N., Del Carmen De Arriba M., García-Barcenilla S., García M., Illera N., Pérez-Conde I., Carretero M. (2020). Beneficial effect of systemic allogeneic adipose derived mesenchymal cells on the clinical, inflammatory and immunologic status of a patient with recessive dystrophic epidermolysis Bullosa: A case report. Front. Med..

[76] Riedl J., Popp C., Eide C., Ebens C., Tolar J. (2021). Mesenchymal stromal cells in wound healing applications: Role of the secretome, targeted delivery and impact on recessive dystrophic epidermolysis bullosa treatment. Cytotherapy.

[77] Lee S.E., Lee S.-J., Kim S.-E., Kim K., Cho B., Roh K., Kim S.-C. (2021). Intravenous allogeneic umbilical cord blood–derived mesenchymal stem cell therapy in recessive dystrophic epidermolysis bullosa patients. JCI Insight.

[78] Rashidghamat E., Kadiyirire T., Ayis S., Petrof G., Liu L., Pullabhatla V., Ainali C., Guy A., Aristodemou S., McMillan J.R. (2019). Phase I/II open-label trial of intravenous allogeneic mesenchymal stromal cell therapy in adults with recessive dystrophic epidermolysis bullosa. J. Am. Acad. Dermatol..

[79] Kühl T., Mezger M., Hausser I., Handgretinger R., Bruckner-Tuderman L., Nyström A. (2015). High local concentrations of intradermal MSCs restore skin integrity and facilitate wound healing in dystrophic epidermolysis Bullosa. Mol. Ther..

[80] Kiritsi D., Dieter K., Niebergall-Roth E., Fluhr S., Daniele C., Esterlechner J., Sadeghi S., Ballikaya S., Erdinger L., Schauer F. (2021). Clinical trial of ABCB5+ mesenchymal stem cells for recessive dystrophic epidermolysis bullosa. JCI Insight.

[81] Różycka-Baczyńska A.M., Stepaniec I.M., Warzycha M., Zdolińska-Malinowska I., Oldak T., Rozwadowska N., Kolanowski T.J. (2024). Development of a novel gene expression panel for the characterization of MSCs for increased biological safety. J. Appl. Genet..

[82] Alsultan A., Farge D., Kili S., Forte M., Weiss D.J., Grignon F., Boelens J.J. (2024). International Society for Cell and Gene Therapy Clinical Translation Committee recommendations on mesenchymal stromal cells in graft-versus-host disease: Easy manufacturing is faced with standardizing and commercialization challenges. Cytotherapy.

[83] Sukmana B.I., Margiana R., Almajidi Y.Q., Almalki S.G., Hjazi A., Shahab S., Romero-Parra R.M., Alazbjee A.A.A., Alkhayyat A., John V. (2023). Supporting wound healing by mesenchymal stem cells (MSCs) therapy in combination with scaffold, hydrogel, and matrix; State of the art. Pathol. Res. Pract..

[84] Liu C., Pei M., Li Q., Zhang Y. (2022). Decellularized extracellular matrix mediates tissue construction and regeneration. Front. Med..

